# Aqua­trinitrato[2,4,6-tris­(pyridin-2-yl)-1,3,5-triazine]neodymium(III) dihydrate

**DOI:** 10.1107/S1600536811014589

**Published:** 2011-05-07

**Authors:** Jin Zhou, Gan-Xiao Lu, Yan-Guang Zhang, Dan-Yi Wei

**Affiliations:** a State Key Laboratory Base of Novel Functional Materials and Preparation Science, Faculty of Materials Science and Chemical Engineering, Ningbo University, Ningbo, Zhejiang 315211, People’s Republic of China

## Abstract

In the title compound, [Nd(NO_3_)_3_(C_18_H_12_N_6_)(H_2_O)]·2H_2_O, the Nd^3+^ ion is in a distorted bicapped square-anti­prismatic geometry formed by three N atoms from the 2,4,6-tris­(pyridin-2-yl)-1,3,5-triazine (TPTZ) ligand, six O atoms from the three nitrate anions and one O atom from the aqua ligand. The mol­ecules are linked by O—H⋯O and O—H⋯N hydrogen bonds. Two types of π–π stacking inter­actions occur between the TPTZ ligands of adjacent complexes [centroid-to-centroid distances = 3.760 (4) and 3.870 (3) Å].

## Related literature

For general background, see: Feng *et al.* (2010 ▶); Long *et al.* (2006 ▶).
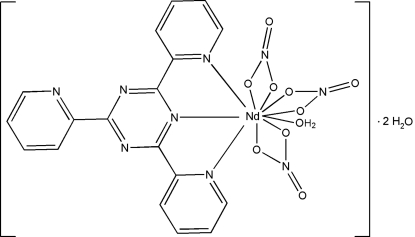


## Experimental

### 

#### Crystal data

[Nd(NO_3_)_3_(C_18_H_12_N_6_)(H_2_O)]·2H_2_O
*M* *_r_* = 696.65
Triclinic, 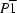

*a* = 9.5799 (5) Å
*b* = 11.9688 (7) Å
*c* = 12.5711 (6) Å
α = 115.376 (5)°
β = 102.611 (4)°
γ = 94.659 (5)°
*V* = 1245.68 (11) Å^3^
*Z* = 2
Mo *K*α radiation
μ = 2.17 mm^−1^
*T* = 293 K
0.29 × 0.24 × 0.09 mm

#### Data collection

Rigaku R-AXIS RAPID diffractometer
Absorption correction: multi-scan (*ABSCOR*; Higashi, 1995 ▶) *T*_min_ = 0.785, *T*_max_ = 1.000
9838 measured reflections
5928 independent reflections
5090 reflections with *I* > 2σ(*I*)
*R*_int_ = 0.026

#### Refinement

*R*[*F* ^2^ > 2σ(*F* ^2^)] = 0.034
*wR*(*F* ^2^) = 0.084
*S* = 1.09
5928 reflections
361 parameters
H-atom parameters constrained
Δρ_max_ = 1.62 e Å^−3^
Δρ_min_ = −0.98 e Å^−3^

### 

Data collection: *RAPID-AUTO* (Rigaku, 1998 ▶); cell refinement: *RAPID-AUTO*; data reduction: *CrystalStructure* (Rigaku/MSC, 2002 ▶); program(s) used to solve structure: *SHELXS97* (Sheldrick, 2008 ▶); program(s) used to refine structure: *SHELXL97* (Sheldrick, 2008 ▶); molecular graphics: *ORTEPII* (Johnson, 1976 ▶); software used to prepare material for publication: *SHELXL97*.

## Supplementary Material

Crystal structure: contains datablocks global, I. DOI: 10.1107/S1600536811014589/ff2007sup1.cif

Structure factors: contains datablocks I. DOI: 10.1107/S1600536811014589/ff2007Isup2.hkl

Additional supplementary materials:  crystallographic information; 3D view; checkCIF report

## Figures and Tables

**Table 1:** Selected bond lengths (Å)

Nd—O10	2.437 (3)
Nd—O1	2.502 (4)
Nd—O5	2.514 (3)
Nd—O8	2.514 (4)
Nd—O4	2.551 (4)
Nd—O7	2.564 (3)
Nd—N2	2.590 (3)
Nd—O2	2.615 (4)
Nd—N3	2.641 (4)
Nd—N1	2.659 (4)

**Table 2:** Hydrogen-bond geometry (Å, °)

*D*—H⋯*A*	*D*—H	H⋯*A*	*D*⋯*A*	*D*—H⋯*A*
O10—H10*B*⋯O5^i^	0.84	2.03	2.819 (5)	156
O10—H10*A*⋯O11	0.84	1.84	2.636 (7)	158
O11—H11*B*⋯O12^ii^	0.84	1.95	2.785 (8)	172
O11—H11*A*⋯O12^iii^	0.84	2.04	2.876 (7)	175
O12—H12*A*⋯N6	0.84	1.99	2.788 (6)	159
O12—H12*B*⋯O3^iv^	0.84	2.18	2.925 (7)	148

Symmetry codes: (i) 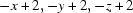; (ii) 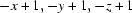; (iii) 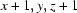; (iv) 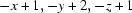.

**Fig. 1.**
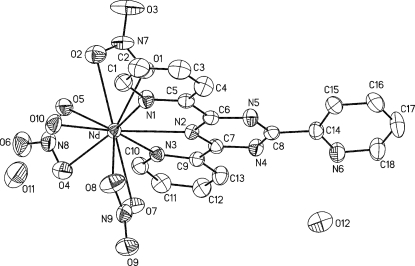
ORTEP plot of complex molecule of (I). Displacement ellipsoids are drawn at the 45% probability level. H atoms were omitted for clarity.

**Fig. 2.**
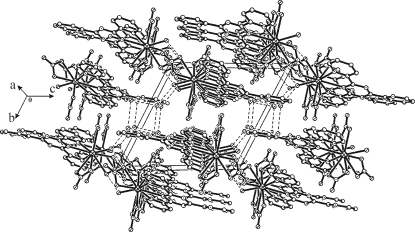
Crystal structure of (I). H atoms were omitted for clarity, hydrogen bonds are drawn as dashed line.

## supplementary crystallographic information

### Comment

Lanthanide complexes earned the interest due to the large coordination spheres, unique magnetic and fluorescence properties of lanthanide ions [Feng *et al.*, 2010; Long *et al.*, 2006]. Herein, the title compound was synthesized and its crystal structure is reported.

In the title compound, [Nd(C_18_H_12_N_6_)(NO_3_)_3_(H_2_O)].2H_2_O (I), the Nd^3+^ ion is coordinated by three N atoms from TPTZ ligand, six O atoms from three nitrate anions and one O atoms from water molecules to form a distorted bicapped square-antiprismatic geometry (Fig. 1). The average bond lengths of Nd—O and Nd—N are 2.5290 (1) Å and 2.6309 (1) Å, respectively. The complexes are interlinked by O—H···O hydrogen bonds between coordinated water molecules and uncoordinated water molecules, O—H···N hydrogen bonds between N6 and lattice water molecules (Fig. 2), and two types of π–π stacking interactions are between the TPTZ ligand of adjacent complexes [centroid–centroid distances = 3.760 (4) Å, 3.870 (3) Å].

### Experimental

All reagents are commercially available and of analytical grade. NdNO_3_.nH_2_O 0.0661 g and TPTZ 0.0312 g (0.1 mmol) were dissolved in 10 ml DMF in beaker. The beaker were put into wide mouth bottle, in which were placed 10 ml of ethanol. The wide mouth bottle was sealed and standed at room temperature. The colorless crystal were obtained after several months.

### Refinement

H atoms bonded to C were placed geometrically and treated as riding, with *U*_iso_(H) = 1.2*U*_eq_(C). The water-bound H atoms were located at difference Fourier maps, and refined as riding with O—H = 0.84 Å and *U*_iso_(H) = 1.5*U*_eq_(O).

### Crystal data

**Table:**

[Nd(NO_3_)_3_(C_18_H_12_N_6_)(H_2_O)]·2H_2_O	*Z* = 2
*M**_r_* = 696.65	*F*(000) = 690
Triclinic, *P*1	*D*_x_ = 1.857 Mg m^−^^3^
Hall symbol: -P 1	Mo *K*α radiation, λ = 0.71073 Å
*a* = 9.5799 (5) Å	Cell parameters from 5280 reflections
*b* = 11.9688 (7) Å	θ = 3.1–29.7°
*c* = 12.5711 (6) Å	µ = 2.17 mm^−^^1^
α = 115.376 (5)°	*T* = 293 K
β = 102.611 (4)°	Plate, colourless
γ = 94.659 (5)°	0.29 × 0.24 × 0.09 mm
*V* = 1245.68 (11) Å^3^	

### Data collection

**Table:**

Rigaku R-AXIS RAPID diffractometer	5928 independent reflections
Radiation source: fine-focus sealed tube	5090 reflections with *I* > 2σ(*I*)
graphite	*R*_int_ = 0.026
ω scans	θ_max_ = 29.8°, θ_min_ = 3.1°
Absorption correction: multi-scan (*ABSCOR*; Higashi, 1995)	*h* = −9→12
*T*_min_ = 0.785, *T*_max_ = 1.000	*k* = −15→14
9838 measured reflections	*l* = −15→16

### Refinement

**Table:**

Refinement on *F*^2^	Primary atom site location: structure-invariant direct methods
Least-squares matrix: full	Secondary atom site location: difference Fourier map
*R*[*F*^2^ > 2σ(*F*^2^)] = 0.034	Hydrogen site location: inferred from neighbouring sites
*wR*(*F*^2^) = 0.084	H-atom parameters constrained
*S* = 1.09	*w* = 1/[σ^2^(*F*_o_^2^) + (0.0117*P*)^2^ + 3.9883*P*] where *P* = (*F*_o_^2^ + 2*F*_c_^2^)/3
5928 reflections	(Δ/σ)_max_ = 0.001
361 parameters	Δρ_max_ = 1.62 e Å^−^^3^
0 restraints	Δρ_min_ = −0.98 e Å^−^^3^

### Special details

**Table:**

Geometry. All esds (except the esd in the dihedral angle between two l.s. planes) are estimated using the full covariance matrix. The cell esds are taken into account individually in the estimation of esds in distances, angles and torsion angles; correlations between esds in cell parameters are only used when they are defined by crystal symmetry. An approximate (isotropic) treatment of cell esds is used for estimating esds involving l.s. planes.
Refinement. Refinement of F^2^ against ALL reflections. The weighted R-factor wR and goodness of fit S are based on F^2^, conventional R-factors R are based on F, with F set to zero for negative F^2^. The threshold expression of F^2^ > 2sigma(F^2^) is used only for calculating R-factors(gt) etc. and is not relevant to the choice of reflections for refinement. R-factors based on F^2^ are statistically about twice as large as those based on F, and R- factors based on ALL data will be even larger.

### Fractional atomic coordinates and isotropic or equivalent isotropic displacement parameters (Å^2^)

**Table:**

	*x*	*y*	*z*	*U*_iso_*/*U*_eq_	
Nd	0.76592 (3)	0.84166 (2)	0.73838 (2)	0.02711 (7)	
N1	0.9322 (4)	0.7339 (4)	0.5944 (3)	0.0361 (9)	
N2	0.6692 (4)	0.7758 (3)	0.5065 (3)	0.0279 (8)	
N3	0.5257 (4)	0.9142 (3)	0.6656 (3)	0.0299 (8)	
N4	0.4875 (4)	0.7688 (4)	0.3410 (3)	0.0337 (8)	
N5	0.6974 (4)	0.6837 (4)	0.3067 (3)	0.0344 (9)	
C15	0.6178 (6)	0.6582 (5)	0.0673 (4)	0.0430 (12)	
H15A	0.7139	0.6562	0.1003	0.052*	
N7	0.9674 (4)	1.0566 (4)	0.7547 (4)	0.0408 (10)	
N8	0.7168 (4)	0.9971 (5)	0.9803 (4)	0.0427 (10)	
N9	0.5837 (5)	0.5887 (4)	0.6550 (3)	0.0419 (10)	
O1	0.8338 (4)	1.0220 (4)	0.6964 (3)	0.0500 (9)	
O2	1.0163 (4)	0.9934 (4)	0.8075 (3)	0.0474 (9)	
O3	1.0430 (5)	1.1464 (4)	0.7595 (5)	0.0786 (15)	
O4	0.6517 (4)	0.8858 (4)	0.9142 (3)	0.0517 (9)	
O5	0.8056 (4)	1.0388 (3)	0.9365 (3)	0.0440 (8)	
O6	0.6986 (5)	1.0642 (4)	1.0778 (3)	0.0656 (12)	
O7	0.5274 (4)	0.6821 (3)	0.6647 (3)	0.0491 (9)	
O8	0.7219 (4)	0.6100 (3)	0.6787 (4)	0.0572 (10)	
O9	0.5138 (5)	0.4864 (4)	0.6241 (4)	0.0674 (12)	
O10	0.9433 (4)	0.7963 (3)	0.8783 (3)	0.0468 (9)	
H10B	1.0087	0.8606	0.9257	0.070*	
H10A	0.9328	0.7462	0.9079	0.070*	
O11	0.9437 (6)	0.6039 (4)	0.9285 (5)	0.0938 (18)	
H11B	0.9245	0.5247	0.8942	0.141*	
H11A	0.9968	0.6244	0.9993	0.141*	
O12	0.1290 (4)	0.6569 (4)	0.1647 (4)	0.0613 (11)	
H12A	0.2151	0.6633	0.1599	0.092*	
H12B	0.0989	0.7163	0.2138	0.092*	
C1	1.0632 (5)	0.7158 (5)	0.6386 (5)	0.0428 (12)	
H1	1.0989	0.7463	0.7230	0.051*	
C2	1.1485 (6)	0.6540 (5)	0.5655 (5)	0.0500 (14)	
H2	1.2382	0.6413	0.5999	0.060*	
C3	1.0993 (6)	0.6118 (5)	0.4419 (5)	0.0497 (13)	
H3	1.1555	0.5708	0.3910	0.060*	
C4	0.9655 (6)	0.6307 (5)	0.3939 (5)	0.0447 (12)	
H4	0.9298	0.6029	0.3099	0.054*	
C5	0.8850 (5)	0.6915 (4)	0.4722 (4)	0.0306 (9)	
C6	0.7427 (5)	0.7185 (4)	0.4261 (4)	0.0294 (9)	
C7	0.5426 (5)	0.8000 (4)	0.4596 (4)	0.0290 (9)	
C8	0.5690 (5)	0.7109 (4)	0.2688 (4)	0.0309 (9)	
C9	0.4623 (5)	0.8728 (4)	0.5452 (4)	0.0292 (9)	
C10	0.4532 (5)	0.9796 (5)	0.7425 (4)	0.0370 (11)	
H10	0.4958	1.0104	0.8262	0.044*	
C11	0.3170 (5)	1.0044 (5)	0.7047 (5)	0.0423 (12)	
H11	0.2691	1.0489	0.7618	0.051*	
C12	0.2550 (5)	0.9625 (5)	0.5825 (5)	0.0422 (12)	
H12	0.1645	0.9788	0.5548	0.051*	
C13	0.3288 (5)	0.8952 (5)	0.5006 (4)	0.0390 (11)	
H13	0.2890	0.8656	0.4168	0.047*	
C14	0.5196 (5)	0.6815 (4)	0.1374 (4)	0.0366 (11)	
N6	0.3811 (5)	0.6860 (4)	0.0949 (4)	0.0445 (10)	
C18	0.3380 (6)	0.6651 (6)	−0.0216 (5)	0.0534 (15)	
H18	0.2412	0.6660	−0.0537	0.064*	
C17	0.4276 (7)	0.6426 (6)	−0.0965 (5)	0.0608 (17)	
H17	0.3928	0.6303	−0.1766	0.073*	
C16	0.5698 (7)	0.6383 (6)	−0.0512 (5)	0.0530 (14)	
H16	0.6328	0.6221	−0.1005	0.064*	

### Atomic displacement parameters (Å^2^)

**Table:**

	*U*^11^	*U*^22^	*U*^33^	*U*^12^	*U*^13^	*U*^23^
Nd	0.02569 (12)	0.02828 (12)	0.02657 (12)	0.00553 (9)	0.00467 (8)	0.01311 (9)
N1	0.032 (2)	0.044 (2)	0.035 (2)	0.0142 (17)	0.0090 (16)	0.0199 (18)
N2	0.0273 (19)	0.0285 (19)	0.0259 (18)	0.0053 (15)	0.0061 (14)	0.0114 (15)
N3	0.0289 (19)	0.034 (2)	0.0307 (19)	0.0083 (16)	0.0105 (15)	0.0170 (16)
N4	0.033 (2)	0.038 (2)	0.029 (2)	0.0084 (17)	0.0063 (15)	0.0152 (17)
N5	0.037 (2)	0.037 (2)	0.029 (2)	0.0107 (17)	0.0102 (16)	0.0142 (17)
C15	0.044 (3)	0.050 (3)	0.036 (3)	0.006 (2)	0.016 (2)	0.019 (2)
N7	0.034 (2)	0.027 (2)	0.064 (3)	0.0037 (17)	0.021 (2)	0.020 (2)
N8	0.038 (2)	0.060 (3)	0.032 (2)	0.015 (2)	0.0082 (17)	0.023 (2)
N9	0.049 (3)	0.038 (2)	0.029 (2)	0.003 (2)	0.0071 (17)	0.0107 (18)
O1	0.043 (2)	0.051 (2)	0.059 (2)	−0.0021 (17)	−0.0011 (17)	0.0373 (19)
O2	0.0329 (19)	0.050 (2)	0.064 (2)	0.0122 (16)	0.0114 (16)	0.0313 (19)
O3	0.053 (3)	0.052 (3)	0.152 (5)	0.009 (2)	0.047 (3)	0.058 (3)
O4	0.049 (2)	0.054 (2)	0.052 (2)	0.0007 (19)	0.0158 (17)	0.025 (2)
O5	0.050 (2)	0.043 (2)	0.0332 (18)	−0.0009 (16)	0.0143 (15)	0.0129 (16)
O6	0.065 (3)	0.089 (3)	0.037 (2)	0.020 (2)	0.0236 (19)	0.020 (2)
O7	0.036 (2)	0.040 (2)	0.069 (3)	0.0028 (16)	0.0130 (17)	0.0248 (19)
O8	0.042 (2)	0.036 (2)	0.084 (3)	0.0082 (17)	0.0094 (19)	0.023 (2)
O9	0.087 (3)	0.035 (2)	0.063 (3)	−0.020 (2)	0.010 (2)	0.0170 (19)
O10	0.047 (2)	0.046 (2)	0.041 (2)	0.0050 (17)	−0.0052 (15)	0.0237 (17)
O11	0.115 (4)	0.056 (3)	0.091 (4)	−0.011 (3)	−0.029 (3)	0.049 (3)
O12	0.051 (2)	0.050 (2)	0.074 (3)	0.0105 (19)	0.019 (2)	0.020 (2)
C1	0.034 (3)	0.051 (3)	0.046 (3)	0.008 (2)	0.009 (2)	0.025 (2)
C2	0.032 (3)	0.059 (4)	0.069 (4)	0.018 (3)	0.014 (2)	0.037 (3)
C3	0.043 (3)	0.050 (3)	0.065 (4)	0.021 (3)	0.028 (3)	0.026 (3)
C4	0.041 (3)	0.049 (3)	0.045 (3)	0.019 (2)	0.019 (2)	0.017 (2)
C5	0.030 (2)	0.027 (2)	0.035 (2)	0.0055 (18)	0.0106 (18)	0.0146 (19)
C6	0.030 (2)	0.026 (2)	0.032 (2)	0.0041 (17)	0.0107 (17)	0.0129 (18)
C7	0.028 (2)	0.031 (2)	0.031 (2)	0.0068 (18)	0.0089 (17)	0.0178 (19)
C8	0.034 (2)	0.025 (2)	0.030 (2)	−0.0002 (18)	0.0083 (17)	0.0107 (18)
C9	0.027 (2)	0.030 (2)	0.032 (2)	0.0063 (17)	0.0074 (17)	0.0164 (19)
C10	0.038 (3)	0.040 (3)	0.036 (3)	0.011 (2)	0.014 (2)	0.018 (2)
C11	0.040 (3)	0.045 (3)	0.048 (3)	0.020 (2)	0.021 (2)	0.021 (2)
C12	0.032 (3)	0.051 (3)	0.049 (3)	0.019 (2)	0.012 (2)	0.027 (3)
C13	0.034 (3)	0.047 (3)	0.037 (3)	0.014 (2)	0.0076 (19)	0.020 (2)
C14	0.045 (3)	0.035 (3)	0.028 (2)	0.006 (2)	0.0084 (19)	0.013 (2)
N6	0.045 (3)	0.055 (3)	0.034 (2)	0.011 (2)	0.0089 (18)	0.022 (2)
C18	0.053 (3)	0.070 (4)	0.038 (3)	0.015 (3)	0.006 (2)	0.028 (3)
C17	0.076 (4)	0.081 (5)	0.032 (3)	0.023 (4)	0.015 (3)	0.030 (3)
C16	0.062 (4)	0.061 (4)	0.043 (3)	0.007 (3)	0.023 (3)	0.027 (3)

### Geometric parameters (Å, °)

**Table:**

Nd—O10	2.437 (3)	N9—O8	1.271 (5)
Nd—O1	2.502 (4)	O10—H10B	0.8399
Nd—O5	2.514 (3)	O10—H10A	0.8400
Nd—O8	2.514 (4)	O11—H11B	0.8400
Nd—O4	2.551 (4)	O11—H11A	0.8400
Nd—O7	2.564 (3)	O12—H12A	0.8405
Nd—N2	2.590 (3)	O12—H12B	0.8396
Nd—O2	2.615 (4)	C1—C2	1.380 (7)
Nd—N3	2.641 (4)	C1—H1	0.9300
Nd—N1	2.659 (4)	C2—C3	1.363 (8)
Nd—N8	2.975 (4)	C2—H2	0.9300
Nd—N9	2.989 (4)	C3—C4	1.373 (7)
N1—C1	1.332 (6)	C3—H3	0.9300
N1—C5	1.346 (6)	C4—C5	1.379 (6)
N2—C6	1.335 (5)	C4—H4	0.9300
N2—C7	1.338 (5)	C5—C6	1.478 (6)
N3—C10	1.328 (6)	C7—C9	1.475 (6)
N3—C9	1.346 (5)	C8—C14	1.486 (6)
N4—C8	1.331 (6)	C9—C13	1.376 (6)
N4—C7	1.337 (5)	C10—C11	1.389 (7)
N5—C6	1.328 (5)	C10—H10	0.9300
N5—C8	1.338 (6)	C11—C12	1.363 (7)
C15—C16	1.366 (7)	C11—H11	0.9300
C15—C14	1.390 (7)	C12—C13	1.382 (7)
C15—H15A	0.9300	C12—H12	0.9300
N7—O3	1.218 (5)	C13—H13	0.9300
N7—O2	1.259 (5)	C14—N6	1.332 (6)
N7—O1	1.261 (5)	N6—C18	1.336 (6)
N8—O6	1.204 (5)	C18—C17	1.367 (8)
N8—O4	1.243 (6)	C18—H18	0.9300
N8—O5	1.283 (5)	C17—C16	1.372 (8)
N9—O9	1.202 (5)	C17—H17	0.9300
N9—O7	1.251 (5)	C16—H16	0.9300
			
O10—Nd—O1	120.22 (12)	O3—N7—O1	121.3 (5)
O10—Nd—O5	79.00 (12)	O2—N7—O1	116.0 (4)
O1—Nd—O5	73.81 (12)	O3—N7—Nd	176.6 (4)
O10—Nd—O8	69.07 (12)	O2—N7—Nd	60.6 (2)
O1—Nd—O8	151.33 (13)	O1—N7—Nd	55.4 (2)
O5—Nd—O8	134.08 (13)	O6—N8—O4	124.0 (5)
O10—Nd—O4	77.59 (13)	O6—N8—O5	120.9 (5)
O1—Nd—O4	117.41 (13)	O4—N8—O5	115.1 (4)
O5—Nd—O4	49.78 (11)	O6—N8—Nd	177.5 (4)
O8—Nd—O4	90.63 (13)	O4—N8—Nd	58.3 (2)
O10—Nd—O7	107.37 (12)	O5—N8—Nd	56.8 (2)
O1—Nd—O7	132.39 (12)	O9—N9—O7	123.3 (5)
O5—Nd—O7	116.65 (12)	O9—N9—O8	122.2 (5)
O8—Nd—O7	49.34 (12)	O7—N9—O8	114.4 (4)
O4—Nd—O7	69.68 (12)	O9—N9—Nd	177.9 (4)
O10—Nd—N2	140.25 (12)	O7—N9—Nd	58.3 (2)
O1—Nd—N2	69.56 (11)	O8—N9—Nd	56.1 (2)
O5—Nd—N2	136.67 (11)	N7—O1—Nd	100.0 (3)
O8—Nd—N2	86.55 (13)	N7—O2—Nd	94.6 (3)
O4—Nd—N2	135.54 (12)	N8—O4—Nd	97.2 (3)
O7—Nd—N2	75.24 (12)	N8—O5—Nd	97.9 (3)
O10—Nd—O2	71.11 (12)	N9—O7—Nd	97.1 (3)
O1—Nd—O2	49.32 (11)	N9—O8—Nd	99.0 (3)
O5—Nd—O2	66.19 (11)	Nd—O10—H10B	110.4
O8—Nd—O2	127.57 (12)	Nd—O10—H10A	130.4
O4—Nd—O2	112.49 (12)	H10B—O10—H10A	112.7
O7—Nd—O2	176.68 (12)	H11B—O11—H11A	104.5
N2—Nd—O2	103.96 (11)	H12A—O12—H12B	123.0
O10—Nd—N3	155.50 (12)	N1—C1—C2	123.3 (5)
O1—Nd—N3	71.23 (11)	N1—C1—H1	118.4
O5—Nd—N3	84.52 (11)	C2—C1—H1	118.4
O8—Nd—N3	112.20 (12)	C3—C2—C1	119.0 (5)
O4—Nd—N3	77.94 (12)	C3—C2—H2	120.5
O7—Nd—N3	64.41 (12)	C1—C2—H2	120.5
N2—Nd—N3	62.49 (11)	C2—C3—C4	119.0 (5)
O2—Nd—N3	118.21 (11)	C2—C3—H3	120.5
O10—Nd—N1	80.40 (12)	C4—C3—H3	120.5
O1—Nd—N1	82.91 (13)	C3—C4—C5	118.9 (5)
O5—Nd—N1	134.92 (12)	C3—C4—H4	120.5
O8—Nd—N1	71.64 (13)	C5—C4—H4	120.5
O4—Nd—N1	155.61 (12)	N1—C5—C4	122.8 (4)
O7—Nd—N1	107.65 (12)	N1—C5—C6	115.9 (4)
N2—Nd—N1	61.87 (11)	C4—C5—C6	121.2 (4)
O2—Nd—N1	69.30 (12)	N5—C6—N2	124.7 (4)
N3—Nd—N1	123.80 (11)	N5—C6—C5	117.1 (4)
O10—Nd—N8	77.08 (12)	N2—C6—C5	118.2 (4)
O1—Nd—N8	96.09 (13)	N4—C7—N2	124.5 (4)
O5—Nd—N8	25.30 (11)	N4—C7—C9	117.4 (4)
O8—Nd—N8	112.58 (14)	N2—C7—C9	118.0 (4)
O4—Nd—N8	24.49 (12)	N4—C8—N5	124.9 (4)
O7—Nd—N8	92.81 (12)	N4—C8—C14	118.1 (4)
N2—Nd—N8	142.56 (11)	N5—C8—C14	116.9 (4)
O2—Nd—N8	89.72 (12)	N3—C9—C13	123.1 (4)
N3—Nd—N8	80.26 (11)	N3—C9—C7	116.9 (4)
N1—Nd—N8	153.26 (11)	C13—C9—C7	120.0 (4)
O10—Nd—N9	87.86 (12)	N3—C10—C11	123.6 (4)
O1—Nd—N9	149.12 (11)	N3—C10—H10	118.2
O5—Nd—N9	128.36 (11)	C11—C10—H10	118.2
O8—Nd—N9	24.82 (11)	C12—C11—C10	118.8 (4)
O4—Nd—N9	78.69 (12)	C12—C11—H11	120.6
O7—Nd—N9	24.53 (11)	C10—C11—H11	120.6
N2—Nd—N9	80.62 (11)	C11—C12—C13	118.8 (4)
O2—Nd—N9	152.39 (12)	C11—C12—H12	120.6
N3—Nd—N9	88.31 (12)	C13—C12—H12	120.6
N1—Nd—N9	90.18 (12)	C9—C13—C12	118.9 (4)
N8—Nd—N9	103.12 (12)	C9—C13—H13	120.6
C1—N1—C5	117.0 (4)	C12—C13—H13	120.6
C1—N1—Nd	121.9 (3)	N6—C14—C15	123.3 (4)
C5—N1—Nd	121.0 (3)	N6—C14—C8	116.3 (4)
C6—N2—C7	115.4 (4)	C15—C14—C8	120.3 (4)
C6—N2—Nd	122.8 (3)	C14—N6—C18	116.6 (4)
C7—N2—Nd	121.8 (3)	N6—C18—C17	124.0 (5)
C10—N3—C9	116.8 (4)	N6—C18—H18	118.0
C10—N3—Nd	122.5 (3)	C17—C18—H18	118.0
C9—N3—Nd	120.2 (3)	C18—C17—C16	118.6 (5)
C8—N4—C7	115.2 (4)	C18—C17—H17	120.7
C6—N5—C8	115.3 (4)	C16—C17—H17	120.7
C16—C15—C14	118.4 (5)	C15—C16—C17	119.2 (5)
C16—C15—H15A	120.8	C15—C16—H16	120.4
C14—C15—H15A	120.8	C17—C16—H16	120.4
O3—N7—O2	122.7 (5)		

### Hydrogen-bond geometry (Å, °)

**Table:**

*D*—H···*A*	*D*—H	H···*A*	*D*···*A*	*D*—H···*A*
O10—H10B···O5^i^	0.84	2.03	2.819 (5)	156
O10—H10A···O11	0.84	1.84	2.636 (7)	158
O11—H11B···O12^ii^	0.84	1.95	2.785 (8)	172
O11—H11A···O12^iii^	0.84	2.04	2.876 (7)	175
O12—H12A···N6	0.84	1.99	2.788 (6)	159
O12—H12B···O3^iv^	0.84	2.18	2.925 (7)	148

Symmetry codes: (i) −*x*+2, −*y*+2, −*z*+2; (ii) −*x*+1, −*y*+1, −*z*+1; (iii) *x*+1, *y*, *z*+1; (iv) −*x*+1, −*y*+2, −*z*+1.
